# Trace elements in glucometabolic disorders: an update

**DOI:** 10.1186/1758-5996-2-70

**Published:** 2010-12-19

**Authors:** Nicolas Wiernsperger, JeanRobert Rapin

**Affiliations:** 1 INSERM U870, INSA Lyon, 6 Bld J. Capelle, F-69621 Villeurbanne (France; 2 Faculté de Médecine/Pharmacie, Université de Bourgogne, 3 Bld jeanne d'Arc, F-21000 Dijon (France

## Abstract

Many trace elements, among which metals, are indispensable for proper functioning of a myriad of biochemical reactions, more particularly as enzyme cofactors. This is particularly true for the vast set of processes involved in regulation of glucose homeostasis, being it in glucose metabolism itself or in hormonal control, especially insulin. The role and importance of trace elements such as chromium, zinc, selenium, lithium and vanadium are much less evident and subjected to chronic debate. This review updates our actual knowledge concerning these five trace elements. A careful survey of the literature shows that while theoretical postulates from some key roles of these elements had led to real hopes for therapy of insulin resistance and diabetes, the limited experience based on available data indicates that beneficial effects and use of most of them are subjected to caution, given the narrow window between safe and unsafe doses. Clear therapeutic benefit in these pathologies is presently doubtful but some data indicate that these metals may have a clinical interest in patients presenting deficiencies in individual metal levels. The same holds true for an association of some trace elements such as chromium or zinc with oral antidiabetics. However, this area is essentially unexplored in adequate clinical trials, which are worth being performed.

## Introduction

Many trace elements, among which metals, involved as cofactors in myriads of biochemical-especially-enzymatic reactions. As such they play cardinal roles in many physiological processes, in particular immunity and metabolism. A good example to illustrate their important contribution is magnesium: low magnesium levels have been associated with increased type 2 diabetes [1,2], whereas controversy exists about the importance of hypomagnesaemia in prediabetic states [3].

Trace elements have been identified for long time as potential candidates for improving metabolic disorders like prediabetes [insulin resistance, obesity, metabolic syndrome] or diabetes. In parallel with increasing comprehension of cellular and biochemical mechanisms leading to-or aggravating- these metabolic disorders, identifying the cellular targets and sites of action of trace elements has reactivated interest in their therapeutic potential. Activation of insulin receptor signalling (chromium), antioxidant properties (selenium, zinc) or inhibition of phosphatases (vanadium) thus appeared promising in view of the key importance of these processes in glucose homeostasis and insulin sensitivity. Indeed insulin receptor/postreceptor signalling defects are considered to underlie glycemic dysregulation and insulin resistance, although the precise causal defects must still be unravelled [4,5]. Prediabetic states, and even more so frank diabetes, are characterized by inflammation [cytokines] and oxidative stress, due to disruption of the equilibrium between production of free radicals and their scavenging by multiple antioxidant systems [6-8]. Moreover, these mechanisms may be involved in concert in the pathogenesis of insulin resistance and accompanying pathologies [9].

The present review updates our actual state of knowledge about those five trace elements (chromium, zinc, selenium, vanadium, lithium)which have a therapeutic potential in insulin resistance and diabetes, describing their mechanisms of action and their efficacy in both animal and human investigations.

## Chromium

### General aspects

The importance of chromium (Cr) for glucose metabolic regulation has been seen in clinical states of relatively severe Cr deficiency, characterized by impaired glucose tolerance, fasting hyperglycaemia and eventually lipid disorders [10,11]. Severe deficiencies are mostly limited to parenteral nutrition but analysis of diet plans currently in use in United States reveals a lack of Cr in many diets, potentially linked to various chronic diseases [12]. The safe and adequate daily intake of Cr was considered to be in the range 50-200 mg. However, now 30 mg/d seems a newly admitted value. Cr is found in relatively high amounts in barley [13]. Cr is poorly absorbed and only toxic in high doses, depending on the milieu, the tissues and the valence of the metal: hexavalent Cr is highly toxic whereas the in vivo toxicity of trivalent Cr is debated [10,14]. After a period of intense utilization, Cr seems to be less popular nowadays, as recently reviewed [15]. Studies performed over the last 20 years in both animals and humans have been largely criticized and whether Cr is indeed an essential element has been questioned [15,16].

Cr has no effect in healthy individuals and, because these are not Cr-deficient, there is no need for supplementation. Cr has been administered and tested in various forms such as Cr-chloride, Cr-nicotinate, Cr-proprionate, Cr-histidinate or Cr-picolinate, the latter being by far the best investigated form because of its solubility in water at neutral pH. However in acidic milieu it hydrolyzes and it has been shown that only about 1% of absorbed Cr is found in the bloodstream [15]. Most of the studies published about biological effects of Cr have indeed used Cr-picolinate. Recently new forms aimed at improving Cr bioavailability have been investigated: a complex of Cr and D-phenylalanine [17], Cr-dinicocysteinate [18] and a combination of Cr and biotin [19].

### Mechanisms of action

Several mechanistic studies showed improvements in insulin receptor/postreceptor signalling, leading to increased glucose transport by enhanced activity of the hormone-sensitive GLUT-4 transporters. These effects can be due to the receptor itself, its control mechanisms or its immediate environment in the cell membrane. Cr-picolinate was shown to induce various effects: increase in AMPKinase and p38 MAPkinase activities [20], increase in receptor phosphorylation [21] and increase in insulin receptor mRNA (in addition to mRNA for GLUT-4, glycogen synthase and UCP3)[22]. Increased synthesis of IGF receptors, able to functionally replace failing insulin receptors, was also described [23]. Changes in the environment of insulin receptors were shown as reductions in plasma membrane cholesterol [24,25], leading to improved cytoskeletal functioning and enhanced insulin sensitivity. Decreases in TNFα [26,27], resistin [28], interleukin-6, CRP [27] as well as increases in vitamin C or adiponectin [29] were all positive mechanisms to reduce insulin resistance by Cr.

Finally, one should mention that Cr can bind directly to insulin, in particular to insulin dimers [30], thereby possibly stabilizing the hormone structure and/or modifying its receptor binding. Interestingly the Cr binding to insulin receptors occurs on a sequence of 3 aminoacids which are identical to those described for GTF decades ago (see below).

### Biological effects

The appearance of glucose intolerance or even fasting hyperglycaemia in demonstrated cases of Cr deficiency have rapidly led to investigations on the potential of Cr supplementation in insulin resistance and diabetes. Furthermore, diabetic patients have about 1/3 less Cr in various biological samples [31-33]. The reasons for this have recently been discussed [15]. While it was shown that inorganic Cr was poorly active, Mertz and coworkers identified as early as 1955 a complex found in brewer's yeast, liver and kidney which was able to stimulate glucose transport in the presence-but not in absence- of insulin and which contained Cr. They termed it GTF [glucose tolerance factor]. Its activity required insulin and was dependent on its Cr content, ideally 4 ions/molecule, while 8 ions/molecule revealed toxic [34,35]. GTF (MW about 1500) appeared to be a mixture of Cr, nicotinic acid and the 3 aminoacids constitutive of glutathione [36]. Replacement of Cr by other metals did not reproduce GTF biological activity. It increased insulin efficacy in adipocytes [35,37] at relatively high concentrations but no effects was found in diabetic patients despite demonstrable increases in Cr content of various tissues [38]. Presently the GTF story is highly criticized because of missing informations and doubtful technical purification procedures [15]. The GTF story continued with description of a similar structure termed LMWCr.

As for many therapeutic trials, concern must be raised as to the concentrations used. Especially in vitro and in animals studies are usually performed with irrelevant concentrations of the active compound, leading to so-called "pharmacological" effects. Accordingly these data should be viewed with appropriate caution. In vitro many beneficial effects of Cr salts have been reported for insulin resistance or diabetes; Cr chloride increased glucose transport in fat and muscle cells as well as in cardiomyocytes [17,25,35]. This was partly explained by higher translocation of Glut-4 transporters [20]. It also decreased oxidative stress, glycosylation and lipid peroxidation in erythrocytes and monocytes under hyperglycaemic conditions [26,39-42].

In animals Cr-picolinate reduced AUC for glucose in type1 diabetic rats [37]. Insulin resistance improved in high fat/low Cr diet-fed rats [43], in sucrose-fed rats [44], in Zucker diabetic fatty rats [29], in GK rats [45,46], in JCR-LA corpulent rats [47] and in db/db mice [48]. A study in obese Zucker rats failed to report improvements [49].

In addition to positive effects on glucose and insulin metabolic profiles, some studies also reported reduction in triglycerides [37] and stimulation of β-oxidation [50].

In contrast to animal studies, human studies look much more controversial. This may be due to large differences in Cr administration protocols. Positive reports with Cr exist in type2 diabetics treated with sulfonylureas [51] or in combination with either biotin [52] or vitamins C and E [53]. Many human studies failed to see improvements in diabetes, as evidenced by reviews and meta-analyses [54-56]. It is likely that patient selection is cardinal [57] and that Cr supplementation is primarily of interest in patients suffering from clearcut Cr deficiency if the aim is to reduce hyperglycaemia [58] or as an adjuvant to already-existing treatment with oral antidiabetics. Changes in blood lipids in humans were not conclusive.

Based on mechanistic hypotheses (see below) the best application might be insulin resistance (prediabetes, metabolic syndrome), although this is also controversial: reduction in insulin resistance was observed in diabetics [53,59] but also in obese women suffering polycystic ovary syndrome [60]. Another study in metabolic syndrome found no effect, however [61].

## Zinc

### General aspects

Zinc (Zn) is an essential micronutrient which has an important role in the functioning of hundreds of enzymes [62], in insulin metabolism and acts as an efficient antioxidant [63,64]. Consequently many observations relate to Zn deficiency, although clinical signs of deficiency are quite modest. Zn is found mainly in cereals, meat, seafood and dairy products [65]. Although usual intakes of Zn are harmless, the range between safe and unsafe is relatively narrow [66]. Because Zn has no storage form, there is need for a constant supply and Zn availability to cells is particularly well regulated, albeit poorly understood [67,68]. Zn absorption can be reduced by iron or inflammatory bowel diseases. It is transported across cell membranes via two families of transporters: ZnT which stimulate Zn efflux and Zip which stimulate its influx [69]. Over 20 transporters are identified and most are intracellular [70]. The implication and regulation of Zn transporters in chronic diseases has been reviewed [71,72]. Concerning metabolic diseases (insulin resistance, metabolic syndrome, diabetes), Zn is considered important mainly because 1) it plays a major role in the stabilization of insulin hexamers and the pancreatic storage of the hormone [73] and 2) it is an efficient antioxidant [74], while oxidative stress is considered to be a main component in initiation and progression of insulin resistance and diabetes [7,75].

Severe Zn deficiency is not frequent but concerns have nevertheless been raised about Zn levels in diabetic patients because of increased excretion due to polyuria. Whereas some studies have reported Zn deficiency in type 2 diabetes [31,76-78], others failed to find significant differences with healthy subjects [79,80]. In type 1 diabetes, Zn deficiency is expected to increase the pancreatic damage [81]. A recent report described reduced levels of Zn in obese, insulin resistant subjects [82]. Lower Zn plasma concentrations were found in type 2 diabetics but they were not related to glycemic status or diabetes duration or components of the metabolic syndrome [83]. Interestingly it was reported that diabetic have elevated levels of copper [78,80] and it could be that copper is in fact linked to metabolic syndrome and diabetes [80,84]. Nevertheless, reduced Zn levels in diabetics appear to be related to increased risk for coronary artery disease [77] and mortality [85].

### Mechanisms of action

Several modes of action have been described to explain the improved action of insulin by Zn. It appears that Zn can have direct insulin-like effects, which may be due to inhibition of the important glycogen-regulating enzyme GSK3 [86]. Other mechanisms include stimulation of the postreceptor proteins Akt and PI3-kinase. Zn can also reduce cytokines such as IL-1β as well as NFκB [87,88]. Zn induces metallothionein synthesis, whereby Zn may have indirect efficacy [87]. Finally it has been suggested that the important Zn concentrations for its action are the intracellular -rather than plasma- levels [89].

Zn is a remarkable antioxidant: it acts at specific sites where it can compete for iron and copper; it further binds to SH groups in proteins, protecting them from oxidation [90]. Because Zn controls metallothionein expression it is involved in cellular redox regulation [91,92]. Oxidative stress can be corrected by dietary Zn as demonstrated by an elevation of hepatic antioxidant enzymes [93]. In contrast, a recent study showed that in vitro even micromolar concentrations were cytotoxic under prevailing H2O2-induced oxidative stress [94]. The latter finding is in line with many observations on prooxidant potential of antioxidants, according to doses and present situation [95,96].

### Biological effects

Despite the potential interest in Zn in diabetes only some investigations have been published (reviewed in 62). However the conclusions are far from clear. In animals Zn prevented the development of type 1 diabetes induced by alloxan or streptozotocin [87]. In a study on Zn restriction during pregnancy in rats, offsprings presented reduced body weight, increased fat mass and low lean mass and showed reduced insulin response to glucose challenge. Zn supplementation corrected the situation only in females [97]. In db/db mice Zn reduced hyperglycaemia and hyperinsulinaemia [98]. Administration **of **Zn for 3 months reduced proteinuria [99]. Zn restriction increased oxidative stress and the levels of isoprostanes [100], in line with antioxidant effects of Zn. Excessive Zn, on the other hand, increased metabolic syndrome in rats fed a high fat/sucrose diet [101]; this could be reversed by copper and magnesium-enriched poultry egg [102].

High Zn slightly reduced the risk for type 2 diabetes in a prospective study performed in 8300 women and the effect was limited to the Zn-deficient subgroup [103]. Another study, however, reported aggravation of glucose intolerance in Zn-deficient diabetic patients [104]. In microalbuminuric type 2 diabetic patients Zn lowered homocysteine levels while increasing vitamin B12 and folate levels [105]. In obese, non-diabetic subjects insulin sensitivity was improved without changes in leptin levels [106]. However no preventive effect against diabetes development could be found in obese women [107]. Oxidative stress was reduced by Zn in healthy elderly subjects [108].

Some particular forms of Zn have been tested recently. Zn-α2-glycoprotein [ZAG] is an adipokine which stimulates energy expenditure in skeletal muscle and brown adipose tissue, resulting in reductions in body weight, glycaemia, triglycerides and NEFAs as found in ob/ob mice. It increases the pancreatic insulin content and improves the glucose tolerance test [109]. Its levels are lower in obese human subcutaneous and visceral adipose tissue and liver, but interestingly does not appear to be related to insulin resistance [110].

Zn-dithiocarbamate and a complex of Zn with garlic-originated allixin improved diabetes in KK mice [111,112].

## Selenium

### General aspects

Selenium (Se) is relatively well absorbed from diet, better so if it is in an organic form [113]. It acts as an antioxidant in the form of selenoproteins which contain selenocysteins [114]. About 10 different selenoproteins are described, the best known being glutathione peroxidases, thioredoxin reductases and iodothyronine deiodinases [115]. Selenoproteins are also responsible for the transport of Se to tissues [116,117]. Severe Se deficiency is rare, while reduced Se levels are seen in diabetics together with increased oxidative stress. In offsprings of diabetic patients Se correlates inversely with CRP and with insulin resistance if Se levels are below 80 μg/l [118]. Excess of Se results in excessive oxidative stress and inflammation as well as diseases like idiopathic scoliosis in adolescents [119] or amyotrophic lateral sclerosis [120]. Presently a lively debate exists as to the benefit of supranutritional doses of Se in cancer [121].

### Biological effects

In view of the potent antioxidant [122,123] and anti-inflammatory effects of Se and the prominent role played by these disorders in insulin resistance and diabetes, supplementation with Se in such diseases appears worthwhile. Interestingly there are only few studies available.

In vitro Se inhibited hyperglycaemia or hyperinsulinaemia-induced expression of adhesion molecules via reduction in p38MAPkinase [124]. It also reduced NFκB, thereby reducing inflammation, CRP and soluble L-selectin [125].

In type 1 diabetic rats Na-selenite protected mitochondria from oxidative stress [126]. Another study showed a better effect with Se-methionine than with Na-selenate [127], confirming other data [128]. In db/db mice selenate reduced gluconeogenesis and inhibited phosphotyrosine phosphatases by 50% [129]. This effect was attributed to formation of selenite from selenate.

In humans lower levels of sialic acid and triglycerides were reported in young adults having the highest dietary Se intake [130]. Reduction in complement factor 3 [C3] where also reported, which may be beneficial considering the link between C3 and body mass index, skin thickness and triglycerides [131]. However no positive effect was found on diabetes prevention and some data even pointed towards an increased risk [132]. A cross-sectional study in almost 9000 american adults as well as another analysis reported a positive link between high Se levels and diabetes [133,134]. Therefore, as is the case for use of high Se in cancer [135], caution applies to utilization of Se supplementation in diabetes, especially since quite high doses are required to observe beneficial effects [113,129].

## Vanadium

### General aspects

Vanadium (Va) has been known for about one century to possess antidiabetic properties [136]. However only over the past 2 decades have concrete therapeutic applications been performed. In humans it is uncertain whether Va is an essential element and it is poorly absorbed in its inorganic form. In vivo Va is transformed into vanadyl cations via glutathione and complexes with ferritin and transferrin [137]. Indeed Va is known to accumulate in some tissues and concerns about its safety have been raised. Bones, liver, kidney lesions as well as loss of weight and gastrointestinal discomfort are well described side-effects of Va, in particular with inorganic Va, whereas organic Va complexes appear more safe [137,138]. Recent reports on Va toxicity show mitochondrial lesions, manifested as swelling and opening of the membrane transition pore due to oxidative stress [139]. Lung cancer in mice is another recently described complication with Va pentoxide [140].

### Mechanisms of action

Va is usually described as "insulin mimetic", in view of several in vitro experiments looking at insulin receptor signaling. The most evident mechanisms is an inhibition of phosphotyrosine phosphatases, leading to increased phosphorylation of IRS-1, PKB, GSK3 and FOXO1 [141,142]. Inhibition of phosphatases is supposed to result from production of peroxyvanadate [137]. Restoration of glucokinase activity was reported in the liver of Va-treated diabetic rats [143]. An ex vivo study in Va-treated gerbils suggested that Va was potentiating, rather than mimicking, insulin and that the main action was on GLUT-4 trafficking [144]. This could explain its poor effects in insulin resistant, normoglycaemic states [145,146].

### Biological effects

In animals; many positive reports exist showing beneficial effects of various Va salts in mild or severe diabetes. For example, Va sulphate or more elaborated organic complexes such as bis(maltolato)oxovanadium [BMOV] have proven efficacy in both type 1 and type 2 models [144,147]. More recently arylalkylamine derivatives reduced glycaemia in various models, alone [148] or in combination (as low doses) with substrates of semicarbazide-sensitive aminooxidase SSAO [149].

In humans many trials were performed. However, as exposed in a review [150], almost all these trials were short-term and included few patients. Glycaemia-reducing activity was seen at doses of 100 mg/d or more [145,151-153]. No efficacy was found, however, in obese non diabetic subjects [145] or in patients with impaired glucose tolerance [146]. Improvements in insulin-sensitivity were claimed to be due to a hepatic effect rather than to an improvement in peripheral insulin resistance [154]. Recently the newer derivatives BEOV and BMOV have entered clinical phase II investigations [155].

## Lithium

### General aspects

Lithium (Li) is a complex trace element mainly used in neurological disorders and more particularly for treating mood and bipolar disorders. Its clinical management is difficult and Li is likely toxic [156,157].

### Mechanisms of action

The interest in Li is based on its ability to inhibit GSK3 [158,159], a key enzyme regulating tissue glycogen levels [160,161]. Glycogen metabolism, on the other hand, is a particularly important component of glucose homeostasis [162]. GSK3 is now also claimed to be involved in bipolar disorders [163]. Very interestingly the prevalence of metabolic dysregulation and diabetes is highly augmented in bipolar disorders [164,165]. Transient diabetes has been associated with Li withdrawal, pointing to the potential effect of this trace element [166].

### Biological effects

In vitro Li increased both basal and insulin-stimulated glucose transport, as well as glycogen synthesis in soleus muscle of insulin resistant, obese Zucker rats [167]. The effect operated via increasing p38MAPKinase, confirming previous similar findings [168].

In vivo Li paradoxically increased glycaemia when administered intravenously to diabetic rats, as shown by increases in glucagon [169]. Another study showed paradoxical effects of oral Li in rat liver: while glycogen synthase was activated and glycogen phosphorylase was decreased, liver glycogen nevertheless decreased [170].

Altogether these data do not support the use of lithium in diabetes despite a cellular target (GSK3) which is potentially of particular interest.

## Conclusion

As a whole, this review shows that trace element supplementation as monotherapy is of weak efficacy in diabetes and hardly efficacious in prediabetic states. The global outcome in humans is somewhat frustrating in view of the logical good expectations from mechanistic effects of these metals at key steps of glucose and insulin regulatory control [171]. As such, these data nicely illustrate a classical observation, i.e. that caution should be given to extrapolations from in vitro or animal data to real human pathology: the data indeed reveal a clear discrepancy between both types of studies. There is little doubt that at least one should 1) better define adequate oral doses and 2) that patient selection should be much more severe. From existing data it appears that in prediabetic or diabetes, trace elements have no or little beneficial effect if the patients do not present deficiencies. It should be stressed out that for all these metals organic derivatives yielded much better results than inorganic forms, likely because of better absorption. Furthermore they could even induce aggravation of metabolic disorders, in line with known inversions of effects of antioxidants at supraphysiological doses. Alternatively the positive effects of micronutrient supplementation is only observed on surrogate parameters such as blood levels of mediators of inflammation and oxidative stress or indices of insulin sensitivity without accompanying improvement in glycaemic control. Another concern is the relatively narrow range between safe and unsafe doses for oral treatment of human individuals.

Although surprisingly few data are available, our belief is that trace elements are of benefit in certain conditions: individual reports indeed suggest that they are of value in situations of trace element deficiency and possibly also in combination with either other trace elements or antioxidants or oral antidiabetics [172,173]. In a model of moderate type 2 diabetes, we found that chronic treatment for 2 weeks using an association of Zn with metformin reduced fasting glycaemia by 30% over metformin alone, while Zn alone was without effect (Rapin and Wiernsperger, unpublished data). Finally trace elements may also be of particular interest in pre/postnatal periods since deficiencies during pregnancy have negative consequences on several constitutive and metabolic parameters: in rat offsprings chromium restriction of mothers during pregnancy resulted in increased visceral adiposity [174], reduced skeletal muscle development and increased basal glucose uptake [175]. Use of a mineral-restricted diet in female weanling rats for 12 weeks resulted in increased body fat and triacylglycerol in offsprings [176].

## Competing interests

The authors declare that they have no competing interests.

## Authors' contributions

NW wrote the manuscript and performed the literature review. JR revised the manuscript and made modifications of intellectual content. Both authors read and approved the final manuscript.
